# Protective effect of Guanxin Danshen formula on myocardial ischemiareperfusion injury in rats

**DOI:** 10.1590/acb380123

**Published:** 2023-04-21

**Authors:** Lanfang Li, Bo Liu, Min Wang, Jingxue Ye, Guibo Sun

**Affiliations:** 1 Institute of Medicinal Plant Development – Peking Union Medical College and Chinese Academy of Medical Sciences - Beijing, China.

**Keywords:** Myocardial Ischemia, Reperfusion, NLR Family, Pyrin Domain-Containing 3 Protein, Ginsenosides

## Abstract

**Purpose:**

Myocardial ischemia/reperfusion injury (MIRI) leads to myocardial tissue necrosis, which will increase the size of myocardial infarction. The study examined the protective effect and mechanism of the Guanxin Danshen formula (GXDSF) on MIRI in rats.

**Methods:**

MIRI model was performed in rats; rat H9C2 cardiomyocytes were hypoxia-reoxygenated to establish a cell injury model.

**Results:**

The GXDSF significantly reduced myocardial ischemia area, reduced myocardial structural injury, decreased the levels of interleukin (IL-1β, IL-6) in serum, decreased the activity of myocardial enzymes, increased the activity of superoxide dismutase (SOD), and reduced glutathione in rats with MIRI. The GXDSF can reduce the expression of nucleotide- binding oligomerization domain, leucine-rich repeat and pyrin domain containing nod-like receptor family protein 3 (NLRP3), IL-1β, caspase-1, and gasdermin D (GSDMD) in myocardial tissue cells. Salvianolic acid B and notoginsenoside R1 protected H9C2 cardiomyocytes from hypoxia and reoxygenation injury and reduced the levels of tumor necrosis factor α (TNF-α) and IL-6 in the cell supernatant, decreasing the NLRP3, IL-18, IL-1β, caspase-1, and GSDMD expression in H9C2 cardiomyocytes. GXDSF can reduce the myocardial infarction area and alleviate the damage to myocardial structure in rats with MIRI, which may be related to the regulation of the NLRP3.

**Conclusions:**

GXDSF reduces MIRI in rat myocardial infarction injury, improves structural damage in myocardial ischemia injury, and reduces myocardial tissue inflammation and oxidative stress by lowering inflammatory factors and controlling focal cell death signaling pathways.

## Introduction

Coronary atherosclerotic heart disease, also known as coronary heart disease or ischemic heart disease, manifests mainly as myocardial ischemia and dysfunction caused by coronary artery stenosis that supplies blood to the heart^1^^,^ 2. Clinically, coronary artery perfusion can be restored quickly, and the blood supply to the ischemic myocardium can be provided in time to treat coronary heart disease^3^. However, myocardial tissue cell structure, energy metabolism, and electrophysiology are irreversibly damaged due to prolonged ischemia in the early stage^4^. Further, the damage is aggravated after the restoration of blood supply, leading to myocardial ischemia/reperfusion injury (MIRI)^5^. As one of the symptoms of coronary heart disease, MIRI is involved in various pathologies such as inflammatory response and oxidative stress^6^. An aseptic inflammatory response often accompanies MIRI. Pyroptosis is believed to be involved in MIRI^7^, and the activation of the nod-like receptor family protein 3 (NLRP3) increases the size of myocardial infarction^8^^,^ 9. Novel small-molecule inhibitors of the NLRP3 inflammasome reduce infarct size and improve cardiac function in an animal model of myocardial infarction^10^. Primary ischemic injury and subsequent mitochondrial injury lead to the activation of the NLRP3 inflammasome in the heart that induces exocytosis of inflammatory cells in cardiomyocytes, further increasing myocardial injury and infarct size^11^. NLRP3 inhibition alleviates MIRI^10^. When the heart suffers from MIRI, the size and severity of myocardial infarction are positively correlated with caspase-1 and IL-1β levels, suggesting that NLRP3-induced pyroptosis exacerbates MIRI. Inhibiting the expression of the NLRP3 inflammasome or caspase-1 can inhibit cell pyroptosis, reduce cell damage, and slow down disease progression, offering a new approach to treating heart disease^7^.

Traditional Chinese medicine and its compounds have unique advantages in treating coronary heart disease^12^. Our research group has studied Guanxin Danshen formula (GXDSF) for more than 10 years^13^^-^ 15. The formula, a combination of *Salvia miltiorrhiza*, *Panax notoginseng*, and *Dalbergia odorifera*, is the most used Chinese herbal preparation for the prevention and treatment of coronary heart disease^16^. It activates qi, relieves pain, promotes blood circulation, and eliminates blood stasis. It is mainly used for chest tightness, shortness of breath, palpitations, and other conditions. It has a significant curative effect on coronary heart disease, with qi stagnation and blood stasis as the main pathogenesis.

In this study, the Sprague Dawley rat myocardial ischemia/reperfusion model and H9C2 myocardial hypoxia-reoxygenation model were used to explore the protective effect of GXDSF on MIRI both *in vivo* and *in vitro*. Previous studies demonstrated that GXDSF protects the myocardium and treats left ventricular remodeling caused by MIRI. However, the mechanism of its anti-cell pyroptosis effect has not been fully explained. In this study, the effect of GXDSF on improving MIRI and the mechanism of anti-pyrocytosis were studied, providing a theoretical basis for the research and development of GXDSF for the clinical treatment of MIRI.

## Methods

Adult male Sprague Dawley rats were purchased from the Beijing Vital River Laboratory Animal Technology Co., Ltd., Beijing, China. The license number was SCXK (Beijing) 2019-0010. The animals were housed under standard laboratory conditions (temperature of 25 ± 1 °C, humidity of 50 ± 10%, and 12 h photoperiod) and allowed free access to sterile food and water. All experiments were approved by the Laboratory Animal Ethics Committee of the Institute of Medicinal Plant Development, Peking Union Medical College (SLXD-20210722034), and in accordance with the *Guide for the Care and Use of Laboratory Animals* published by the United States National Institutes of Health.

### Cell

H9C2 rat cardiomyocytes were purchased from the Cell Bank of the Chinese Academy of Sciences.

### Materials

GXDSF (20200905) and trimetazidine hydrochloride tablets (2018655) were purchased from Harbin Yerui Pharmaceutical Co., Ltd. and Servier (Tianjin) Pharmaceutical Co., Ltd., respectively. The creatine kinase MB isozyme (CK-MB) kit (020045), aspartate aminotransferase (AST) kit (020013), and lactate dehydrogenase (LDH) kit (020050) were purchased from Zhongnorth Biotechnology Co., Ltd. The superoxide dismutase (SOD) kit (20211119) and the reduced glutathione^17^ determination kit (20211119) were purchased from Nanjing Jiancheng Science Technology Co., Ltd. The rat IL-1 β (BS-E10967R1) and rat tumor necrosis factor α (TNF-α) kit (BS-E12215R1) were purchased from Boshen Biotechnology Co., Ltd. NLRP3 rabbit antibody (A12694), gasdermin D (GSDMD) rabbit antibody (A18281), IL-1β rabbit antibody (A19635), IL-18 rabbit antibody (A16737), caspase-1 rabbit antibody (A0964), and glyceraldehyde-phosphate dehydrogenase (GAPDH) rabbit antibody (AB181602) were purchased from Abcam, United Kingdom.

### Establishment of animal model and administration

Male Sprague Dawley rats (270–280 g) rats were randomly divided into a sham operation group (n = 15) and MIRI model groups: model (0 mg/kg, n = 15), high (216 mg/kg, n = 15), medium (108 mg/kg, n = 15), low (54 mg/kg, n = 15), and trimetazidine (5.4 mg/kg, n = 15) groups. Sham group and ischemia/reperfusion (IR) group were given equal volume solvent in the stomach. The treatment group was given the above drugs intragastmically for 14 days, and the last treatment was completed 60 min before the MIRI model. The rats were anesthetized by intraperitoneal injection of pentobarbital sodium (30 mg/kg). After endotracheal intubation, the rats were resuscitated on a ventilator, then fixed in the supine position and connected to an electrocardiogram. The chest was then opened and the left anterior descending coronary artery was ligated onto a tube with a line 2 mm below the left atrial appendage^18^. Cardiac ischemia began with an elevation of the ST segment of the electrocardiogram and lasted for 30 min. The coronary artery was not lapped in the sham group and all other procedures were the same as in the model group. After ischemia, the heart was perfused for 24 h. After surgery, the ventilator was removed when the rats were able to breathe on their own. They were then placed on a heated blanket until they could walk and then returned to their cage.

### Cell model establishment and drug administration

When the cell fusion rate reached 70–80%, salvianolic acid B (10, 5, 2.5, and 1.25 μmol.L^–1^) was added successively, and notoginsenoside R1 (20, 10, 5, and 2.5 μmol.L^–1^) was added for 24 h. Then, the medium was changed to a sugar-free version. After being hypoxic for 4.5 h in the anaerobic tank, the medium was replaced with a serum-free Dulbecco’s modified eagle medium for reoxygenation for 2 h.

### 2,3, 5-triphenyltetrazole chloride (TTC) staining

The 2% TTC solution was preheated to 37 °C. After blood was collected from the rat’s abdominal aorta, the heart was removed and frozen in liquid nitrogen for 20 s, and the heart was sectioned. Six heart slices were cut from the apex and stained with the TTC solution for 8 min. The tissue was fixed in a tissue fixative solution and photographed 24 h later. The area of myocardial infarction was measured using the ImageJ software.

### Hematoxylin-eosin (HE) staining

Myocardial tissue soaked in tissue fixative solution for more than 24 h was washed with distilled water and soaked in gradient ethanol until complete dehydration and soaked twice in xylene before embedding in paraffin. The embedded tissue was cut into 5 μm slices using a slicer, placed in a baking machine for 60 min, dewaxed with xylene, and stained with hematoxylin. The tissue was placed in 1% hydrochloric acid-ethanol for 30 s and balanced with distilled water. The samples were placed in 1% ammonia for 30 s, rinsed again, stained with eosin, and then rinsed with distilled water. The tissue was dehydrated with gradient ethanol and xylene and fixed with a neutral resin seal. The tissue structures were observed and recorded under a microscope. Observe whether the myocardium structure of the heart is abnormal, whether there is inflammatory cell infiltration, whether there is necrosis of myocardial cells and so on^19^.

### Enzyme-linked immunosorbent assay (ELISA)

The kit reagents were placed in 96-well plates and balanced at room temperature for 20 min. Standard wells were filled successively with a gradient dilution standard. The samples to be tested were placed in the plates’ wells and incubated with horseradish peroxidase in a 37 °C water bath or thermostat for 1 h. After washing the plate, A and B substrates were added and incubated at 37 °C for 15 min away from light. Finally, a stop solution was added to measure each well’s absorbance at 450 nm.

### Blood biochemical

The serum levels of CK-MB, AST, and LDH were detected using an automatic biochemical analyzer.

### Western blot

Whole-cell proteins were isolated using a protein extraction reagent containing a protease inhibitor mixture. After the tissue was lysed, loading buffer was added, and the mixture was boiled in a water bath to denature the proteins. The resulting samples were added to sodium dodecyl sulfate-polyacrylamide gel to separate the proteins; the proteins were transferred to a nitrocellulose membrane. The membrane was blocked with 5% skimmed milk for 2 h, and incubated overnight at 4 °C with the primary antibody solution. The secondary antibody of the corresponding species was added. The film was developed using a developing solution and analyzed with a gel imaging system (Bio-Rad, Hercules, CA, USA). β-actin expression was measured in each sample to verify equal protein loading.

### Statistical analysis

Data were represented as mean ± standard deviation (SD), and performed the normality test (Shapiro–Wilk). All data are normally distributed. For the column diagrams, one-way ANOVA followed by Tukey’s post-hoc test was used for multiple comparisons^19^. The statistically significant difference between the groups was set at P < 0.05.

## Results

### Protective effect of GXDSF on MIRI rats

#### Effect of GXDSF on myocardial infarction area after MIRI rats

**Figure 1:**
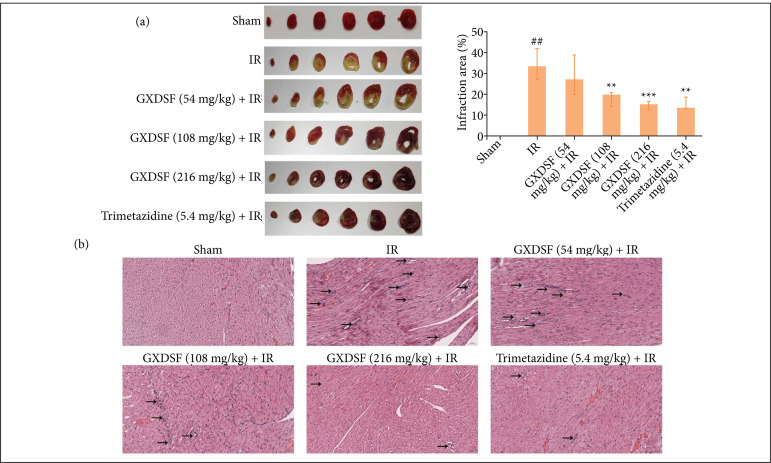
Protective effect of GXDSF on MIRI rats. **(a)** TTC staining was used to evaluate the effect of GXDSF on the area of MIRI in Sprague Dawley rats (n = 8). The data were presented as median (interquartile range), ^###^P < 0.001 vs. sham group. ^**^P < 0.01, ^***^P < 0.001 vs. I/R group; **(b)** Observation of the effect of GXDSF on the myocardial structure of Sprague Dawley rats by HE staining (n = 3).

TTC staining results of rat heart sections are shown in Fig. 1a. No ischemic area was found in the sham group, whereas a large ischemic area was white in the I/R group. Compared with the IR group, the GXDSF group (216, 108, and 54 mg/kg)showed reduced ischemic infarct area in a dose-dependent manner. The high-dose group of GXDSF (216 mg/kg) had the smallest ischemic white area and the best treatment effect, followed by the medium-dose group of GXDSF (108 mg/kg). The positive control trimetazidine group (5.4 mg/kg) had a significantly reduced ischemic area compared with the I/R group. The effect of the GXDSF in the high-dose group (216 mg/kg) was similar to that of the trimetazidine group (5.4 mg/kg) in the positive control, indicating that GXDSF had a significant protective effect on MIRI.

#### Effect of GXDSF on the myocardial structure after MIRI rats

HE staining was used to observe the effect of GXDSF on the myocardial structure of I/R rats (Fig. 1b). In the sham operation group, myocardial cells were slender and neatly arranged, with no obvious abnormalities in the overall myocardial structure. Myocardial injury in the model group was serious, inflammatory cell infiltration was obvious, cell morphology was irregular, and cytoplasm was concentrated. Compared with the model group, the GXDSF (216, 108, and 54 mg/kg)-treated groups showed dose-dependent protection of myocardial structure, alleviated myocardial damage, orderly arrangement of myocardial cells, and significantly reduced myocardial structure damage and inflammatory cell infiltration.

#### Effects of GXDSF on the activities of CK-MB, AST, and LDH in serum of MIRI rats

**Figure 2:**
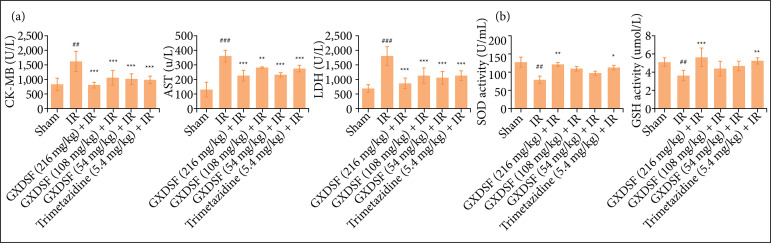
Effects of GXDSF on enzyme activity in MIRI rats. **(a)** Effects of GXDSF on the activities of CK-MB, AST, and LDH in serum of MIRI rats (n = 8); **(b)** Changes of SOD activity and GSH activity in serum of rats after GXDS treatment (n = 8). Values for each group are presented as mean ± SD, ^##^P < 0.01, ^###^P < 0.01 vs. sham group. ^*^P < 0.05, ^**^P < 0.01, ^***^P < 0.001 vs. I/R group.

Compared with the sham operation group, MIRI induced increased levels of AST, LDH, and CK-MB myocardial enzymes, suggesting serious myocardial injury. Compared with the model group, the GXDSF (216, 108, and 54 mg/kg) treatment groups showed significantly reduced elevation of AST, LDH, and CK-MB levels induced by MIRI, alleviated myocardial injury, and heart protection. The positive control trimetazidine group (5.4 mg/kg) also reduced the level of myocardial enzymes induced by MIRI. The effect of the GXDSF high-dose group (216 mg/kg) was similar to that of the trimetazidine group, suggesting that GXDSF can reduce the level of myocardial enzymes to treat MIRI and protect the heart (Fig. 2a).

#### Effects of GXDSF on SOD and GSH activity in MIRI rats

Changes in SOD and GSH activities in the serum of rats were detected using a kit. Compared to the sham operation group, the activities of SOD and GSH in the serum of the model group were significantly decreased. Compared with the model group, SOD activity and GSH activity of the GXDSF high-dose group (216 mg/kg) were significantly increased, and there were no significant differences in SOD and GSH activities between the GXDSF medium- and low-dose groups (108 mg/kg and 54 mg/kg). The activities of SOD and GSH in the trimetazidine group (5.4 mg/kg) were significantly increased. The effect of high-dose GXDSF (216 mg/kg) was similar to that of the trimetazidine group (5.4 mg/kg), indicating that GXDSF can enhance the antioxidant effects of myocardial tissue (Fig. 2b).

### Protective mechanism of GXDSF on MIRI rats

#### Effects of GXDSF on the expression of NLRP3, IL-1β, caspase-1, and GSDMD in MIRI rats

**Figure 3:**
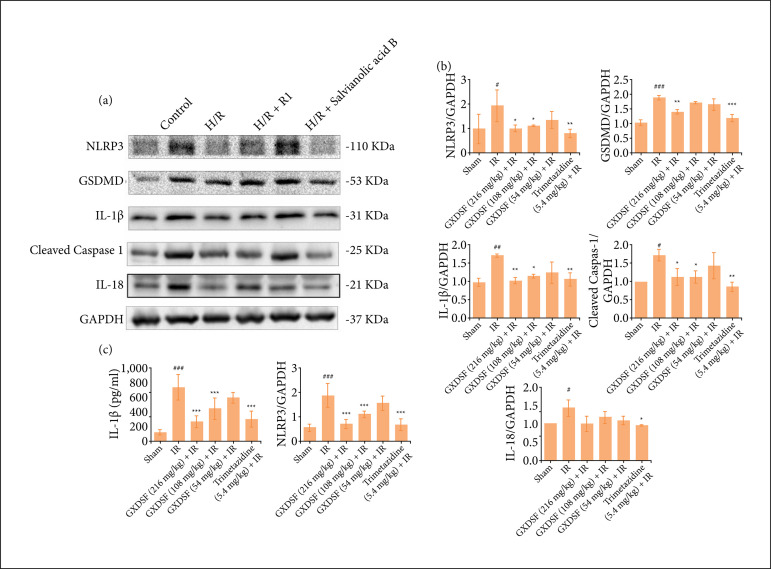
Protective mechanism of GXDSF on MIRI rats. **(a)** Effects of GXDSF on the expression of NLRP3, IL-1β, caspase-1, and GSDMD in MIRI rat (n = 3); **(b)** Changes of IL-1β level and IL-6 level in serum of rats after GXDS treatment; **(c)** The levels of IL- 1β, IL-6 in serum were measured by ELISAValues for each group are presented as mean ± SD (n = 8). ^#^P < 0.05, ^##^P < 0.01, ^###^P < 0.001 vs. sham group. ^*^P < 0.05, ^**^P < 0.01, ^***^P < 0.001 vs. I/R group.

Western blot was used to detect the expressions of NLRP3, IL-1β, caspase-1, and GSDMD in I/R rat heart tissue (Fig. 3a,b). Compared with the sham operation group, the protein expression levels of NLRP3, GSDMD, IL-1β, and caspase-1 in the myocardium of the model group were significantly increased. Compared with the model group, the protein expression levels of NLRP3, GSDMD, IL-1β, and caspase-1 in the GXDSF high-dose group (216 mg/kg) and trimetazidine positive group (5.4 mg/kg) were significantly decreased. The protein expression levels of NLRP3, IL-1β, and caspase-1 in the mid-dose group of GXDSF (108 mg/kg) were also significantly decreased, whereas the protein expression level of GSDMD showed a downward trend with no statistical difference. The protein levels of NLRP3, GSDMD, IL-1 β, and caspase-1 in the GXDSF low-dose group (54 mg/kg) showed a downward trend, with no statistical difference.

#### Effects of GXDSF on serum IL-1β and IL-6 levels in MIRI rats

An ELISA kit was used to detect the levels of inflammatory factors in the serum of the rats (Fig. 3c). Compared to the sham operation group, the levels of inflammation in the serum of the model group were significantly increased, and the heart muscle was damaged. Compared with the model group, the GXDSF high and medium doses (216 mg/kg and 108 mg/kg)significantly reduced serum IL-1β and IL-6, alleviated myocardial injury, and protected the heart. The positive control trimetazidine group (5.4 mg/kg) significantly reduced the levels of IL-1β and IL-6 in the rats’ serum. The effect of the GXDSF high dose (216 mg/kg) was similar to that of trimetazidine, suggesting that GXDSF can protect the damaged heart by reducing the levels of serum inflammatory factors in MIRI.

#### Protective effects of salvianolic acid B and notoginsenoside R1 on hypoxia and reoxygenation of H9C2 cardiomyocytes

**Figure 4:**
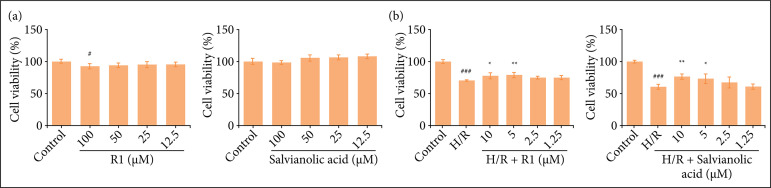
The protective effects of salvianolic acid salvianolic acid B and *P. notoginseng* saponin R1 on H9C2 cells. **(a)** The toxicity of salvianolic acid B and Notoginsenoside R1 on H9C2 cells (n = 6). **(b)** The protective effects of salvianolic acid salvianolic acid B and *P. notoginseng* saponin R1 on H9C2 cells after H/R treatment (n = 6). The data were expressed as the mean ± SD. ^#^P < 0.05, ^###^P < 0.001 vs. control group; ^*^P < 0.05, ^**^P < 0.01, ^***^P < 0.001 vs. H/R group.

The results of the cell counting kit (CCK-8) assay showed that salvianolic acid B was not cytotoxic against H9C2 at concentrations of 100 μmol.L^–1^ and below. Notoginsenoside R1 exhibited cytotoxic effects in H9C2 cells at 100 μmol.L^–1^, as shown in Fig. 4a. Therefore, concentrations below 100 μmol.L^–1^ were used in the subsequent experiments.

H9C2 cardiomyocytes were hypoxic and reoxygenated for 4.5 h and 2 h. The effect of salvianolic acid B and notoginsenoside R1 on the cell survival rate of H9C2 cardiomyocytes after hypoxia and reoxygenation are shown in Fig. 4b. Compared with the control group, the survival rate of H9C2 myocardial cells in the model group was significantly decreased. Compared with the model group, the cell survival rate of the hypoxic/ reoxygenation (H/R) salvianolic acid B (10 and 5 μmol.L^–1^) and of the H/R notoginsenoside R1 (40 and 20 μmol.L^–1^) groups were significantly increased, showing that salvianolic acid B and notoginsenoside R1 protect H9C2 cardiomyocytes from hypoxia and reoxygenation. Salvianolic acid B and notoginsenoside R1 had the best effect at 10 and 20 μmol.L^–1^, respectively.

Effects of salvianolic acid B and notoginsenoside R1 on the expression of NLRP3, IL-1β, IL-18, caspase-1, and GSDMD pyroptosis-related proteins in H9C2 cardiomyocytes

**Figure 5:**
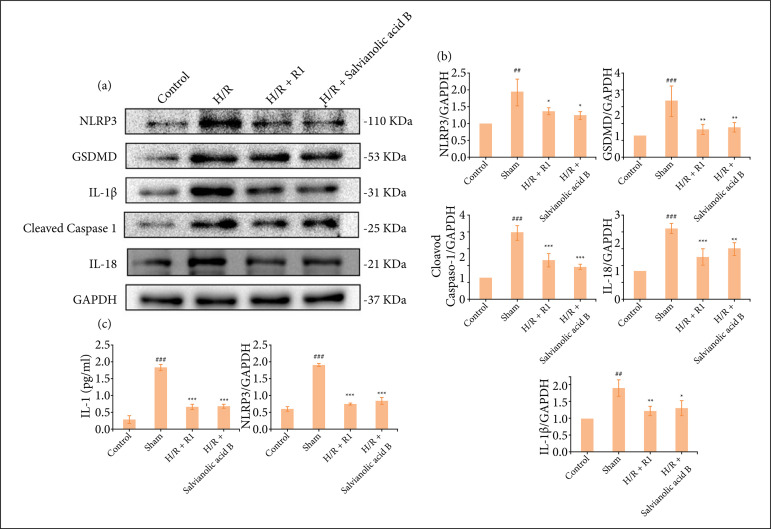
(**a, b**) Western blot detection of the expression of pyroptosis-related proteins (NLRP3, IL-18, Caspase-1, GSDMD) in H/R-treated H9C2 cells by salvianolic acid B and notoginsenoside R1 (n = 3); **(c)** The effects of salvianolic acid salvianolic acid B and *P. notoginseng* saponins R1 on the levels of inflammatory factors TNF-α and IL-1β in the supernatant of H9C2 cells after H/R treatment (n = 6). The data were expressed as the mean ± SD. ^#^P < 0.05, ^##^P < 0.01, ^###^P < 0.001 vs. control group; ^*^P < 0.05, ^**^P < 0.01, ^***^P < 0.001 vs. H/R group.

Western blotting results showed that the protein levels of NLRP3, GSDMD, caspase-1, and IL-18 in H9C2 myocardial cells in the model group were significantly increased compared with the control group. Compared with the model group, the protein expression levels of NLRP3, GSDMD, caspase-1, and IL-18 in the notoginsenoside R1 (20 μmol.L^–1^) and salvianolic acid B (10 μmol.L^–1^) groups were significantly decreased. These results suggest that salvianolic acid B (10 μmol.L^–1^) and notoginsenoside R1 (20 μmol.L^–1^) can regulate pyroptosis-related proteins and protect H9C2 cardiomyocytes from hypoxia and reoxygenation injury (Fig. 5 a,b).

#### Effects of salvianolic acid B and notoginsenoside R1 on the levels of TNF-α and IL-1β inflammatory cytokines in cell supernatants

An ELISA was used to determine the levels of TNF-α and IL-1β inflammatory factors in the cell supernatants of the control, model, H/R salvianolic acid B 10 μmol.L^–1^, and H/R notoginsenoside R1 20 μmol.L^–1^ groups (Fig. 5c). The levels of TNF-α and IL-1β inflammatory factors in the supernatant of the model group were significantly increased. Compared with the model group, the levels of TNF-α and IL-1β inflammatory factors in the cell supernatants of the H/R salvianolic acid B 10 μmol.L^–1^ and H/R notoginsenoside R1 20 μmol.L^–1^ groups were significantly decreased, approaching the levels in the control group. The results suggest that salvianolic acid B and notoginsenoside R1 reduce the levels of inflammatory factors induced by hypoxia and reoxygenation, thus protecting H9C2 myocardial cells from hypoxia and reoxygenation injury.

## Discussion

These results suggest that GXDSF can reduce MIRI in rat myocardial infarction areas. It improves the structure of the myocardial ischemia injury in the rat myocardial damage, reduces myocardial tissue inflammatory cells infiltration and cavitation number, myocardial enzyme activity, and the activity of SOD and GSH. Further, it enhances antioxidant capacity and exhibits a protective role. The protective mechanism of GXDSF may be related to the decrease in IL-1β and IL-6 inflammatory cytokines, NLRP3, caspase-1, and GSDMD protein expression, and regulation of the cell scoria signaling pathway. Our results also show that salvianolic acid B (10 μmol L^–1^) and notoginsenoside R1 (20 μmol L^–1^), two active components of GXDSF, significantly protect H9C2 cardiomyocytes from hypoxia and reoxygenation injury and reduce the levels of TNF-α and IL-1β inflammatory factors in the cell supernatant. The mechanism may be related to the decrease in NLRP3, IL-18, caspase-1, GSDMD protein expression, and cell pyroptosis signaling pathway regulation. Consequently, GXDSF can improve MIRI injury and has a significant protective effect on the heart, which may be related to the regulation of pyroptosis.

In the treatment of coronary heart disease, MIRI is often accompanied by the restoration of the coronary artery blood supply, which seriously harms patients’ health and increases the risk of mortality^20^. Currently, a variety of mechanisms are involved in MIRI^21^^,^ 22. Early mitochondrial damage leads to excess reactive oxygen species production and calcium imbalance. MIRI induces aseptic inflammatory responses^23^. Locally released risk-related molecular patterns lead to the initiation and triggering of NLRP3 and amplify the inflammatory response and cell death by activating caspase 1^24^^,^ 25. In this study, it was found that MIRI can cause myocardial fibrosis, myocardial tissue enlargement, myocardial structural damage, myocardial enzyme leakage, inflammatory cell infiltration, vacuole number in myocardial tissue, oxidative stress, and other phenomena. These results provide new insights into the mechanisms and pathways that regulate MIRI.

GXDSF is a traditional Chinese medicine composed of *S. miltiorrhiza*, *P. notoginseng*, and *D. odorifera* 13. It is mainly used for chest tightness, breathlessness, palpitations, and shortness of breath. At present, there are a variety of derivative drugs, such as Guanxin Danshen tablet and Guanxin Danshen dripping pills, which are often used in the clinical prevention and treatment of coronary heart disease. Previous studies by our group have shown that GXDSF improves cardiovascular diseases and can treat left ventricular remodeling caused by MIRI13-15. Further, it can reduce the size of myocardial infarction in rats with MIRI, improve the damage of myocardial structure, reduce the number of inflammatory cell infiltrates and vacuoles in myocardial tissue, reduce the activity of myocardial enzymes, enhance antioxidant capacity, and play a protective role in the heart.

There are many components of GXDSF. Salvianolic acid B is the active substance in salvianolic acid, and notoginsenoside R1 is the active substance with a higher content in *P. notoginseng* that is easy to extract and separate. According to a previous study, salvianolic acid B and notoginsenoside R1 also have therapeutic effects on cardiac diseases. Therefore, studies on the effects of GXDSF and its active components, salvianolic acid B and notoginsenoside R1, on MIRI and their anti-cell coke death mechanism are helpful to further understand the cardioprotective ability of GXDSF in the treatment of MIRI.

The results of this study showed that salvianolic acid B and notoginsenoside R1 protect H9C2 cardiomyocytes from hypoxia and reoxygenation injury. The mechanism of the decrease in inflammatory factors in the cell supernatant may be related to the decrease in NLRP3, IL-18, caspase-1, and GSDMD protein expression and the regulation of the cell death signaling pathway. These results provide a theoretical basis for the clinical application of GXDSF and an academic reference for its development to treat MIRI.

## Conclusion

GXDSF reduces MIRI in rat myocardial infarction injury, improves structural damage in myocardial ischemia injury, and reduces myocardial tissue inflammation and oxidative stress by lowering inflammatory factors and controlling focal cell death signaling pathways.

## Data Availability

Data will be available upon request.
